# Mutant T4 DNA polymerase for easy cloning and mutagenesis

**DOI:** 10.1371/journal.pone.0211065

**Published:** 2019-01-23

**Authors:** Ruhu Qi, Gottfried Otting

**Affiliations:** Research School of Chemistry, Australian National University, Canberra, ACT, Australia; University of Helsinki, FINLAND

## Abstract

The advent of high-fidelity DNA polymerases that can be used to linearize and amplify whole plasmids by PCR opened the door to greatly simplified cloning and mutagenesis protocols. Commercially available kits work well, but often have been optimized using undisclosed or proprietory components. Here we show that a mutant T4 DNA polymerase (Y320A) with attenuated 3’-exonuclease activity is uniquely suited to generate single-stranded DNA overhangs of uniform length in a more easily controllable manner than the wild-type enzyme, and this can be used to increase the yields of colonies containing correctly modified plasmids in cloning and mutagenesis experiments, which is particularly useful when *E*. *coli* cells are of relatively low competency. Standard protocols using the mutant T4 DNA polymerase are provided for the sequence and ligation independent cloning (SLIC) method and a modified QuikChange method, where the mutant enzyme enhances the yield of correctly mutated plasmid and further suppresses parental plasmid during digestion with DpnI. Single-stranded DNA overhangs generated by the mutant T4 DNA polymerase facilitate subsequent plasmid circularization, annealing and ligation in *E*. *coli*.

## Introduction

Faithful linearization and amplification of whole plasmids by PCR with high-fidelity DNA polymerases present the basis of modern cloning and mutagenesis methods such as sequence and ligation independent cloning (SLIC) [1,2], Gibson assembly [3], and QuikChange (Agilent, La Jolla, CA) [4]. Optimized kits have become available commercially, but often the details of the optimizations have not been described. Having established an in-house protocol for SLIC to save costs for large numbers of reactions, we noticed that the cloning success rate was highly variable and dependent on the batch of T4 DNA polymerase used, indicating that the protocol could be upset by small changes in the 3’-exonuclease activity of the enzyme, which SLIC relies on. This suggested that better control of the enzyme’s activity would render the SLIC protocol more robust. In different published versions of the protocol, the activity of the T4 DNA polymerase was adjusted by adjusting the temperature and duration of treatment [1,2,5–7]. T4 DNA polymerase is a highly active exonuclease, which has been shown to completely digest primers of 17 nucleotide length in 15 seconds at 37 °C in experiments, where a two-fold excess of the enzyme was presented with a short primer/template construct [8]. Such high digestion rates make it difficult to limit the digestion to about 20 nucleotides. In the present work we show that (i) using the wild-type enzyme at a concentration lower than that of the DNA leads to highly non-uniform DNA digestion and (ii) an attenuated mutant offers better control of the exonuclease activity while delivering single-stranded DNA overhangs of uniform length, which is beneficial for cloning with the SLIC method as well as for mutagenesis using QuikChange.

The SLIC method is based on an earlier method, “ligation-independent cloning of PCR products” (LIC-PCR), introduced in 1990 as a method for ligating DNA segments with single-stranded DNA overhangs of an exact number of nucleotides (commonly 12–14 nucleotides) [9,10]. Conveniently, hybridization of 12-nt overhangs generates products that are sufficiently stable for direct transformation of *E*. *coli* cells, where endogenous ligase completes the circularization of the plasmid. Besides its independence from restriction sites, a major attraction of LIC-PCR thus lies in avoiding the need for *in vitro* ligation, which tends to be slow, unreliable and costly. In the original implementation of LIC, the 3’->5’ exonuclease activity of wild-type T4 DNA polymerase was used in the presence of a single nucleotide triphosphate (e.g. dGTP) to expose the complementary strand until the first complementary nucleotide (e.g. cytidine). This approach, however, requires the availability of PCR primers with terminal segments devoid of the nucleotide added in the exonuclease reaction and this sequence restriction is a major impediment, complicating the design of PCR primers [10].

**Fig 1:**
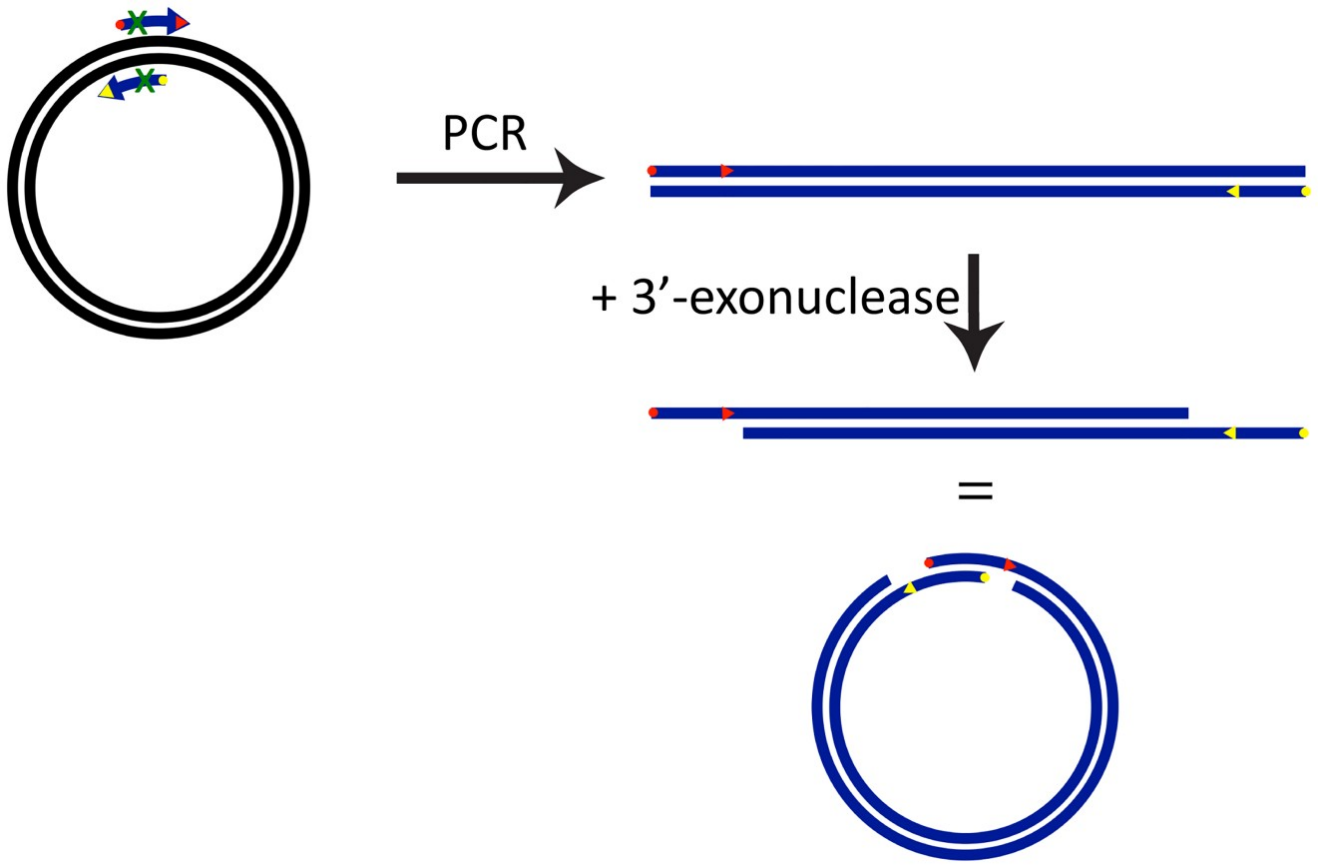
SLIC protocol for cloning. Double-stranded circular plasmid is linearized and amplified by PCR. Alternatively, the vector can be linearized by restriction digestion. The primers used for amplification of the insert must comprise segments that are complementary to the primers used for vector linearization. The start point of matching nucleotide segments are marked by yellow and red points. The PCR products (blue) are combined, treated with 3’-exonuclease and the hybridized mixture is used to transform competent *E*. *coli* cells.

The sequence restrictions of LIC-PCR were overcome by the realization that *E*. *coli* cells can be relied upon to complete the final circular double-stranded vector by ligation, as well as to recreate double-stranded DNA from single-stranded templates. Therefore, the SLIC approach uses the exonuclease activity of T4 DNA polymerase in the absence of any of the four nucleotide triphosphates to generate single-stranded 5’-overhangs of unspecified length, thereby lifting any sequence restrictions for the overhangs [1,2]. The treatment with T4 DNA polymerase is timed and can be stopped by adding dGTP or dCTP [2], by placing on ice [5] or by inactivating the polymerase by heating to 70 °C [1] or the addition of EDTA [11]. The SLIC method has proven highly successful. For example, using 40 base pair (bp) overlaps [2], SLIC was shown to allow the assembly of nine PCR-generated fragments into a vector. A recent comparison of ligation-independent cloning techniques comparing SLIC with polymerase incomplete primer extension (PIPE) cloning [12] and overlap extension cloning (OEC) [13,14] concluded that SLIC provides many more transformants than PIPE and can handle bigger inserts than OEC [15]. Fig 1 shows a graphical illustration of the basic SLIC protocol.

In an analogous way, the cloning protocol introduced by Gibson and co-workers uses a 5’->3’ exonuclease to create single-stranded DNA overhangs, together with a DNA polymerase and *Taq* ligase to generate annealed circular vectors *in vitro* prior to transformation [3]. In contrast to SLIC, Gibson assembly thus avoids possible in-cell digestion of linear DNA. As a drawback, Gibson assembly is more expensive in reagents and requires longer PCR primers, as the complementary DNA overhangs must anneal at higher temperature (50 °C).

To minimize costs, we revisited the SLIC protocol with the aim to improve control of the exonuclease activity of T4 DNA polymerase by using mutant T4 DNA polymerases with attenuated 3’-exonuclease activities. Such mutants have been described previously, including truncated forms and singly or doubly mutated full-length enzymes, for which the exonuclease activity is attenuated to different degrees relative to the wild type [16–18]. To the best of our knowledge, their use for molecular cloning has not been explored. We hypothesized that the attenuated activity of mutant T4 DNA polymerase would facilitate accurate timing of the reaction at 37 °C to generate 5’ single-stranded DNA overhangs of optimal length. The lower activity of the mutants was also expected to allow inactivation by heat (e.g. 20 minutes at 72 °C) without compromising the reaction time by the finite time required to reach the denaturation temperature. (Inactivation by cooling was deemed undesirable as it would lead to non-stringent hybridization.) Our results show that the mutant Y320A of T4 DNA polymerase indeed enhances the success rate of SLIC. Unexpectedly, however, it appears that this effect arises not so much from better control of the digestion reaction as from the greater ease with which a uniform digestion result is achieved by the mutant compared to the wild-type T4 DNA polymerase, which is evidenced in digestion experiments of a 98-basepair DNA duplex. Although the revised SLIC protocol takes somewhat more time than the original version, the improved reliability and yield of correct constructs ultimately accelerates the overall procedure of molecular cloning. We refer to this new method as RQ-SLIC, for being reliable and, therefore, quick.

Generation of single-stranded DNA overhangs with the mutant T4 DNA polymerase can also be used to promote plasmid circularization in the QuikChange protocol for site-directed mutagenesis. QuikChange relies on a pair of primers containing the mutations in a PCR reaction that copies the entire plasmid. To favour hybridization of the primers to the template rather than each other, a modified QuikChange protocol uses primers designed with mutually complementary 5’ segments while the 3’ segments are complementary only to the template [4]. As in SLIC, ligation can be achieved by transformation of *E*. *coli* cells with the unligated plasmid, allowing endogeneous enzymes from the DNA repair system to complete the recircularization and ligation. It has been shown that this step predominantly relies on homologous recombination, which can be promoted by special *E*. *coli* strains [19].

**Fig 2:**
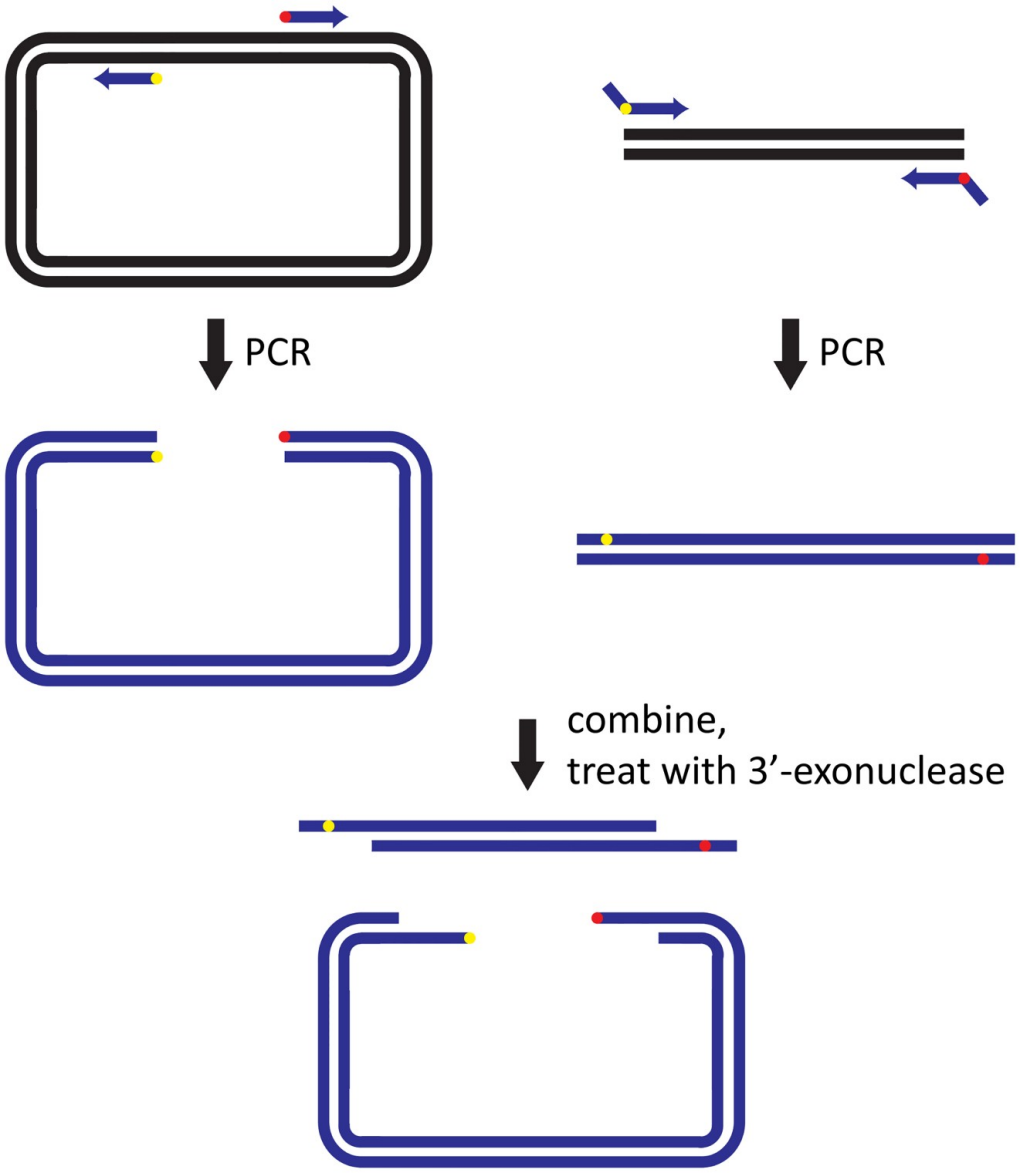
Modified QuikChange mutagenesis scheme using a 3’-exonuclease to generate single-stranded 5’ overhangs. Plasmid DNA is amplified by PCR using a high-fidelity DNA polymerase such as Q5 (New England Biolabs). Forward and reverse primers overlap partially [20]. The red and yellow circles (triangles) mark the 5’ (3’) ends of the primers. The green crosses mark mismatches between the mutation primers and the template DNA. The present work demonstrates the advantage of using an attenuated 3’-exonuclease to generate single-stranded overhangs. Filling in of single-stranded DNA and re-ligation of the circular plasmid is achieved by endogenous *E*. *coli* enzymes following transformation.

Similar to the SLIC protocol, we anticipated that recircularization of the vector can also be promoted by generating matching single-stranded DNA overhangs from the blunt-ended PCR products by limited digestion with the Y320A mutant of T4 DNA polymerase (Fig 2). While the digestion step increased the yield of correctly mutated plasmid as expected, a second benefit arose from prevention of re-ligation of parental plasmid incompletely cut by DpnI, when minimal digestion times were used.

In the following we report the preliminary cloning tests that led us to select the Y320A mutant of T4 DNA polymerase for protocol optimization, an analysis of digestion patterns obtained by the Y320A mutant and the wild-type enzyme, an optimized protocol for RQ-SLIC, and a QuikChange protocol modified by using the mutant T4 DNA polymerase to promote plasmid recircularization and minimize contamination of the final product with parental plasmid.

## Results

### T4 DNA polymerase mutants for improved control of 3’-exonuclease activity

Three mutants of T4 DNA polymerase (T4P) were investigated as suitable substitutes of wild-type T4P, namely the mutants E114A (also referred to as E1), Y320A (also referred to as E2) and a truncation mutant retaining only the 388 N-terminal residues (referred to in the following as N388). The exonuclease activities of the mutants E114A and Y320A have been reported to be reduced, respectively, 10- and 50-fold on double-stranded DNA compared with the wild-type enzyme [18]. The exonuclease activity of the truncation mutant N388 was reported to be similar to that of the wild-type enzyme. We investigated N388 because it was also reported to yield digestion products following a Poisson distribution, at least for single-stranded DNA [17].

### Preliminary testing of mutant T4 DNA polymerases in SLIC

All three mutant proteins were expressed, purified and concentrated. Their performance in the SLIC protocol was tested by counting the number of colonies obtained after transformation of a reaction mixture, where the insert, provided as linear DNA obtained by PCR, and the linearized vector were present in 1:1 molar ratio. The experiments used the 4.7 kb plasmid pETMCSI [21] linearized by digestion with *Nde*I and *Eco*RI. The insert was a 1.2 kb DNA segment containing the N388 gene, which was amplified with the primers 25nt-f and 25nt-r (Table A in S1 File). Both primers share a 25 nt long overlap segment with the ends of the cut plasmid. The exonuclease reaction was performed in T4 DNA ligase buffer (T4 buffer; 50 mM Tris-HCl, pH 7.5, 10 mM MgCl_2_, 10 mM DTT, 1 mM ATP) at 37 °C in a single tube containing 100 ng vector and 25 ng insert DNA in 1:1 molar ratio in a total reaction volume of 20 μL, testing the T4P mutants at 1.6 μM concentration and in 10x, 100x and 1000x dilution with reaction times of 0.5, 1, 2, and 4 h, respectively. The reaction was stopped by heating (72 °C for 20 min) and allowed to cool to room temperature over 5 minutes to allow for overhang annealing. This solution was used to transform 100 μL DH5α cells made chemically competent by the method of Inoue et al. [22]. The cells had a transformation efficiency of 2x10^6^ c.f.u./μg pUC19 plasmid DNA. The transformation mixtures were plated on a LB plate supplemented with 100 μg/mL ampicillin. Digestion with the N388 truncation mutant yielded no colonies at all, also not when the digestion reaction was performed at room temperature, whereas colonies were obtained using the E114A and Y320A mutants. Therefore, only the E114A and Y320A mutants were examined further with the aim to optimize enzyme concentration and reaction time.

For a reaction volume of 20 μL, a concentration of about 200 nM enzyme proved to be optimal for a range of durations of digestion times and lengths of the nucleotide overlap. Digestion with the mutant E114A was tested for durations between 5 and 120 minutes and the Y320A mutant was tested between 15 minutes and 8 h.

**Fig 3:**
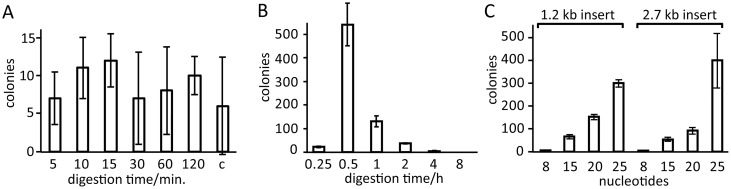
Number of colonies obtained for cloning a 1.2 kb insert into a 4.7 kb linearized vector. DNA was digested at 37 °C with the T4P mutants E114A or Y320A at a final concentration of 0.3 μM. Experiments were performed in triplicate. Vector and insert were used in 1:1 molar ratio. (A) Digestion with E114A for the durations indicated. Column c shows the result of a negative control without T4P treatment, which indicates that the presence of the enzyme made no significant difference. (B) Digestion with Y320A for the durations indicated. (C) Result of digestion with the mutant Y320A for different lengths of insert–vector overlaps (see Table A in S1 File for the primers used to amplify the inserts) and two different sizes of insert (1.2 kb N388 and 2.7 kb wild-type T4P, respectively).

Fig 3A shows that treatment of the reaction mixtures with the mutant E114A resulted in a small number of colonies only, which was comparable to a negative control. In contrast, digestion with the mutant Y320A resulted in a large number of colonies (Fig 3B). Therefore, we continued to investigate the performance of the mutant Y320A (in the following referred to as enzyme E2) for molecular cloning.

### Primer length dependence of cloning

To test suitable lengths of overlaps between insert and vector in the cloning protocol using the E2 enzyme, the number of colonies formed for terminal overlaps between 8 and 25 nucleotides was assessed for cloning an insert into a 4.7 kb pETMCSI vector linearized by double digestion with *Nde*I and *Eco*RI. Vector and insert were treated with E2 enzyme in 20 μL for 1 h at 37 °C. Longer overlaps produced greater yields, which were similar for 1.2 and 2.7 kb inserts (Fig 3C). Sequencing of 36 junctions of 18 randomly selected clones confirmed their correctness with the exception of a single-nucleotide mutation in one of the clones.

### Analysis of E2 exonuclease activity

**Fig 4:**
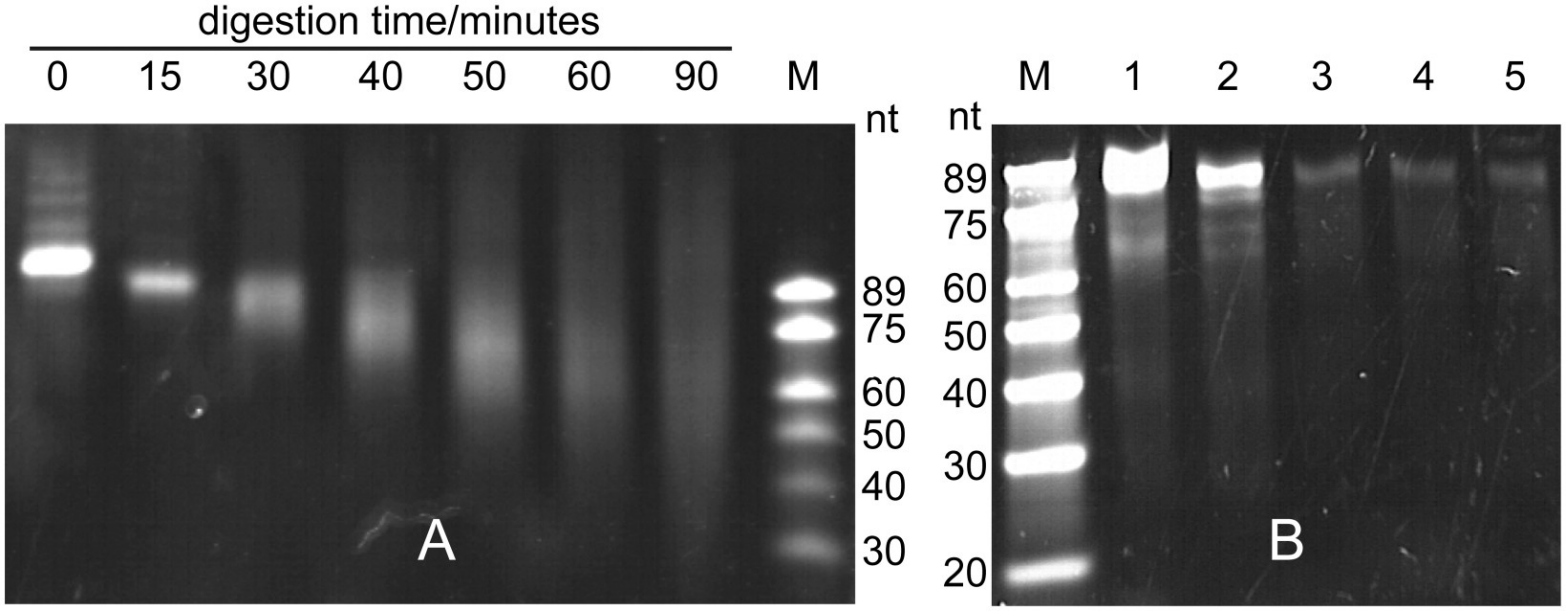
DNA digestion by the E2 enzyme and wild-type T4P. The substrate was a 98-bp double-stranded DNA fragment (Fig A in S1 File) amplified by PCR from pETMCSI with the primers 98bp-f and v-r (Table A in S1 File) and purified by a PCR clean-up kit. The digestion product was purified by phenol extraction, precipitated with ethanol, dried and analysed on a 12% continuous denaturing PAGE run in TBE buffer (89 mM Tris-borate, pH 8.3, 2 mM EDTA) containing 7 M urea. Lane M: DNA markers (number of nucleotides indicated at the side). See Text B in S1 File for more details. (A) Digestion of a 157 nM solution of 98-bp DNA with 1.6 μM E2 at 37 °C. Aliquots were taken at 15 minute intervals as indicated. (B) Digestion of 98-bp DNA (157 nM solution) with wild-type T4P (New England Biolabs) at 16 °C for 30 minutes, using the enzyme at different concentrations. Reactions were terminated by adding 1 μL 10% SDS. Lanes 1–4: T4P used in 100-, 30-, 10-, and 3-fold dilution relative to the amount (5 units or 0.6 μg) used for the reaction in lane 5, which corresponded to a concentration of wild-type T4P of 0.3 μM.

To gain a better understanding of the E2 mutant, we assessed the exonuclease activities of the mutant and wild-type T4P by digesting a 98-bp double-stranded DNA fragment. To account for the very different activities, the digestion with E2 was conducted at 37 °C (i.e. the temperature at which we recommend to use this enzyme in our standard protocols discussed below), while the digestion with wild-type T4P was performed at 16 °C to control its reactivity (even at room temperature, the activity of wild-type T4P is so high that a published SLIC protocol limits the digestion time to 2.5 minutes [5]). Fig 4A shows that 15 minutes digestion with E2 led to a loss of about 10 nucleotides in a fairly uniform manner, suggesting that the enzyme falls off the substrate much faster than it cuts nucleotides. Between 10 and 35 nt were lost after 45 minutes digestion. In contrast, digestion with wild-type T4P produced mixtures of undigested and highly digested DNA. Some undigested DNA persisted even after 30 minutes at about equimolar concentration of T4P and DNA termini (lane 4 of Fig 4B), and undigested DNA became the dominant species when the concentration of T4P was lowered (Fig 4B). Undigested DNA has not been observed in experiments conducted with plasmid fragments generated by restriction digestion and digested by an about 15-fold excess of wild-type T4P for 10 minutes at 10–25 °C [6]. This indicates that the initiation of digestion from blunt-ended DNA presents a barrier for the wild-type enzyme, requiring an excess of enzyme to initiate digestion in a more uniform manner. Owing to its reduced activity, the E2 enzyme can be used in high concentrations at a convenient temperature (37 °C) without losing control over the length of single-stranded overhangs generated due to very fast digestion rates.

### Standard cloning protocol

The following cloning protocol (which we refer to as RQ-SLIC) has become the standard in our laboratory. The protocol uses 20 μL T4 buffer containing 100 ng each of insert and vector backbone, 1 μL each of E2 enzyme (4 μM stock solution) and DpnI (3 units/μL; New England Biolabs). Plasmid is linearized by PCR amplification and purified with a PCR purification kit (Promega, Madison, USA). The E2 digestion is performed at 37 °C and stopped by heating at 72 °C for 20 minutes, followed by standing on the bench for 5 minutes to allow for hybridization of the homologous overhangs generated. Subsequently, the reaction mixture is put on ice and the entire mixture used to transform *E*. *coli* cells.

**Fig 5:**
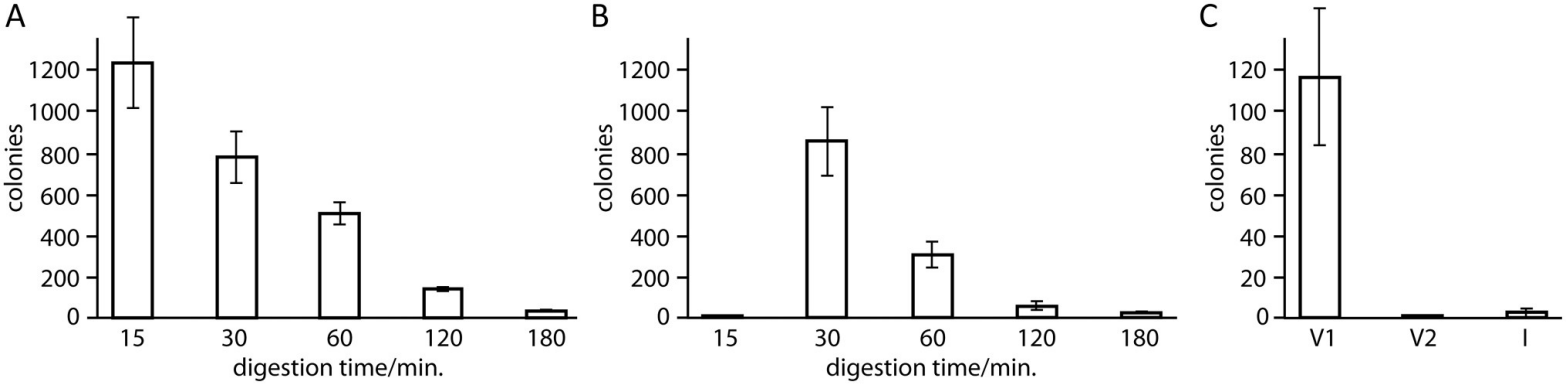
Number of colonies obtained for cloning a 1.2 kb insert into a 4.7 kb vector. The plasmid (pETMCSI) [21] was linearised by PCR using as template (A) undigested and (B) *Nde*I-*Eco*RI double-digested plasmid. Digestions with E2 and DpnI were performed at 37 °C for the durations indicated and all experiments were performed in triplicate. (C) Negative control conducted with vector or insert only, following 60 minutes of E2/DpnI digestion. V1: linearized vector. V2: vector double-digested with *Nde*I and *Eco*RI prior to linearization by PCR. I: insert.

Fig 5A shows the effect of digestion time with E2 in experiments, where the reaction mixture was used to transform 100 μL DH5α cells prepared with a transformation efficiency of 2 x 10^6^ c.f.u./μg of pUC19 vector. A significant number of colonies was obtained following digestion with E2 and DpnI for 15, 30 and 60 minutes, with decreasing numbers for increasing digestion times. This result suggests that the outcome was dominated by parental plasmid in only 15 minutes digestion time, while correct-sized clones are obtained from 30 minutes onwards. This was confirmed by colony PCR of 14 colonies randomly selected from each treatment (Table 1). To minimize any carry-through of parental vector, we repeated the experiment with plasmids that were double-digested with two different restriction enzymes (e.g. *Nde*I and *Eco*RI in the case of pETMCSI) prior to PCR amplification. Fig 5B shows that this resulted in much fewer colonies after 15 minutes digestion, without significantly affecting the yields of correct product following longer digestion times. All clones tested contained the insert. Experience gained in hundreds of reactions performed with the E2 enzyme shows that successful cloning results can be expected for digestion times between 30 minutes to 1 hour. Furthermore, cloning yields proved to be best, if the insert is in excess over the vector. Therefore, the standard cloning protocol uses vector and insert in 1:1 mass ratio (rather than molar ratio).

**Table 1:** Cloning yields obtained with the standard cloning protocol^a^.

	Vector linearized from undigested template			Vector linearized from double-digested template		
E2/DpnI digestion time/min.	15	30	60	15	30	60
Average colony number per reaction	1241	782	508	1	852	304
Number of colonies checked	14	14	14	3^b^	14	14
Colonies with correct inserts (number)	0	11	8	3	14	14
Colonies with correct inserts (%)	0	79	57	100	100	100

^a^ Measured by observation of product of the correct size following colony PCR of the insert.

^b^ To increase the number of colonies for analysis, all three colonies were tested which were obtained in the experiment performed in triplicate.

Double-digestion of the plasmid prior to linearization by PCR is optional, but significantly decreases the chance of parental plasmid passing through the protocol unmodified (Fig 5C). Using digestion times of at least 30 minutes, plasmid templates that were PCR-amplified without prior double-digestion still delivered plasmid with the correct insert in over half of the colonies (Table 1). Therefore, the cloning protocol is applicable to plasmids without restriction sites and does not depend on any particular nucleotide sequence.

We usually perform the digestion with the E2 enzyme in T4 buffer, but the enzyme also works well in water by relying on residual buffering capacity from the 5 mM Tris-HCl (pH 8.0) buffer used for storage of vector and insert DNA. The resulting low-salt condition allows subsequent cell transformation by electroporation without any dilution to avoid high ionic strength. Alternatively, the presence of 10 mM MgCl_2_ in the T4 buffer is fully compatible with the transformation of cells made chemically competent by calcium treatment [22]. Addition of Tris-HCl, ATP and dithiothreitol (DTT) is not required. The E2 enzyme can be stored in a standard buffer (50% glycerol, 10 mM Tris-HCl, pH 7.4, 1 mM DTT, 0.1 mM EDTA, 50 mM KCl, 200 μg/mL BSA) at -20 °C over several years without substantial loss of activity.

### Comparison with established methods

The performance of the RQ-SLIC protocol was compared with the cloning success rates of different published protocols using wild-type T4P by measuring the number of colony forming units obtained with the different digestion protocols. In our hands, the RQ-SLIC protocol produced about two times more colonies than the SLIC method with greater consistency between different trials (Table 2). The RQ-SLIC method thus performs twice as well as SLIC, while offering greater control over the digestion reaction, which is particularly advantageous if a large number of cloning reactions are to be performed in parallel.

**Table 2:** Comparison of the cloning efficiency of RQ-SLIC compared to three established methods based on wild-type T4P^a^.

Method	Original^b^	SLIC^c^	QC^d^	RQ-SLIC^e^
Reference	[1]	[2]	[6]	This work
Trial 1	1	81	2	452
Trial 2	0	143	0	353
Trial 3	0	225	0	394
Average	< 1	150	< 1	400
c.f.u./ng vector	0.0006	1.3	0.007	2.6

^a^ The established methods were conducted following the published protocols exactly, using pETMCSI vector and N388 insert with 25 nt overlaps. The reaction mixtures were stored on ice prior to transformation of 100 μL DH5α cells made competent chemically (2 x 10^6^ c.f.u./μg pUC19 plasmid DNA). The cells were plated on LB medium supplemented with 100 μg/mL ampicillin for overnight growth in a 37 °C incubator.

^b^ Digestion reaction of the original method: 399 ng of vector and 101 ng of insert in 1:1 molar ratio in 33 mM Tris-HCl (pH 7.9), 66 mM potassium acetate, 10 mM magnesium acetate, 0.5 mM DTT, 0.1 mg/mL BSA, 1 unit T4P. This mixture was kept at 37 °C for 2 min, heated to 70 °C for 10 min, kept at 37 °C for another 2 h, followed by the addition of 1 μL 2 mM dNTP, 1 μL 10 mM DTT and 2 units T4P and incubation at 30 °C for 30 min.

^c^ 500 ng vector and insert digested separately, each in 20 μL 1x T4 buffer with 0.5 units T4P at 22 °C for 30 min. The reaction was stopped by adding 1 μL 10 mM dCTP and leaving on ice. Subsequently, a total of 150 ng of vector and insert in 1:1 molar ratio were annealed during 30 min at 37 °C in 10 μL T4 buffer.

^d^ 50 ng vector and 50 ng insert DNA were mixed in 20 μL T4 buffer at 25 °C for 5 min. The reaction was stopped by storing on ice.

^e^ Following the standard cloning protocol described in the present work.

### Standard protocol for site-directed point mutagenesis

The E2 enzyme can be used to improve not only cloning, but also mutagenesis protocols such as the modified QuikChange mutagenesis approach, which relies on a homologous recombination mechanism in *E*. *coli* to re-circularize plasmids containing homologous ends [19]. To assess the potential benefit of controlled E2 digestion of the ends of linearized plasmid prior to transformation of *E*. *coli*, we performed 18 different site-directed mutagenesis reactions with and without E2 digestion using the standard protocol (see below and Fig 2). On average, digestion delivered about five times more colonies while reducing the chance of parental plasmid passing through. This indicates that generation of single-stranded DNA overhangs is beneficial in the same way as in the SLIC protocol. We have successfully applied the standard mutagenesis protocol described below to over 100 different nucleotide sequences.

The protocol starts with linearization of plasmid DNA by PCR. To 200 ng of purified PCR products in 20 μL in T4 buffer are added 1 μL of 4 μM (0.4 mg/mL) stock solution of E2 enzyme and 1 μL of DpnI restriction enzyme stock (New England Biolabs). The mixture is incubated for 0.5–1 h at 37 °C. The reaction is stopped by heating to 72 °C for 20 min, then cooled on bench for 5 min and transferred to ice. The entire reaction mixture is used to transform 100 μL chemically competent cells by the standard heat shock procedure of Green and Sambrook [23].

In a recent university course performed by an undergraduate student class, students introduced additional mutations into two mutant versions of a gene for *p*-cyanophenylalanyl-tRNA synthetase [24] in the expression plasmid pETMCSIII [21] (see Table A in S1 File for the primers used). The final reaction mixture was used to transform DH5α cells, which were plated on LB medium supplemented with 100 μg/mL ampicillin and incubated overnight at 37 °C. On average, each transformation yielded 300–500 colonies. Sequencing of two randomly picked colonies per plate with the PET3 forward and PET4 reverse sequencing primers (Table A in S1 File) showed correct sequences for both colonies in 11 out of 18 cases and one of the colonies contained the correct sequence in the remaining 7 cases. Unsuccessful sequencing results arose from failure to sequence (2 cases), a single nt deletion (1 case) and absence of mutation (4 cases).

Digestion with DpnI reduces the chances of unmodified parental plasmid passing through the protocol. Interestingly, treatment with DpnI after E2 digestion allows more parental plasmid to appear in the final colonies than simultaneous treatment of the DNA with DpnI and E2. This suggests that in-cell re-ligation of DpnI-digested DNA can occur following transformation, if the treatment with DpnI is kept short (0.5–1 h in our protocol). When DpnI is present already during the digestion with E2, cuts made by DpnI are digested further by the E2 enzyme, making subsequent re-ligation of parental plasmid difficult.

### Primer design

In our hands, primers designed for a 15 nt primer–primer overlap already produce reliable results in SLIC and mutagenesis experiments, if the melting temperature of the overlap is at least 45 °C or, better, 50–55 °C. Good results are obtained, when the 3’ ends of the primers following the mismatch produce a melting temperature with the template ten degrees higher than the primer–primer overlap region to ensure preferential primer–template association during PCR amplification.

The SLIC protocol tolerates some mismatch between the melting temperatures at either end of the insert. For example, double digestion of the pETMCSI vector by *Nde*I and *Eco*RI produces an AT-rich end at the *Nde*I site and a GC-rich end at the *Eco*RI site. Although the melting temperatures of 25 nt overlap sequences at these two ends of the template differ by about 20 degrees, this does not prevent successful cloning (Fig 3C).

## Discussion

The present work introduces the mutant Y320A of T4 DNA polymerase (E2 enzyme) for cloning and site-directed mutagenesis. Owing to its slower 3’-exonuclease rate, the E2 enzyme can be used in large excess relative to the DNA to generate uniform single-stranded DNA overhangs without losing control over the digestion rate, which is important for, e.g., manual execution of multiple cloning experiments in parallel. In our hands and compared with conventional restriction–ligation methods, the RQ-SLIC protocol is at least as reliable while providing significant savings in time and cost. Incubation with the E2 enzyme and subsequent heat inactivation can conveniently be performed on a PCR thermocycler.

Wild-type T4P is known to drop off its DNA substrate easily, e.g. after having digested two or three nucleotides [8,25]. Therefore the different digestion pattern observed for the E2 enzyme and wild-type T4P cannot be attributed to different processivity of the exonuclease activity. Instead, the difficulty to initiate digestion of blunt-ended DNA with dilute solutions of T4P (Fig 4B) indicates that creating a 5’ overhang from blunt-ended DNA is a rate-limiting step. In order to obtain a uniform digestion rate, it is thus important to use the exonuclease in sufficiently large excess. This notion is supported by digestion experiments conducted with plasmid fragments produced by restriction digestion, where uniform digestion rates (with a digestion rate of about 20 nucleotides per minute at 25 °C) were observed using a 15-fold molar excess of T4P over DNA fragments [6].

Optimizing the reaction conditions for controlled digestion by wild-type T4P is complicated by different affinities of the enzyme to single-stranded, double-stranded, and primer/template DNA, as well as enzyme dimerization on the DNA [26]. An early experiment of the digestion of short primer/template DNA constructs using an about two-fold excess of T4P reported complete primer digestion in 15 seconds at 37 °C [8], whereas a similar experiment conducted with about three-fold lower DNA and enzyme concentrations yielded an average digestion rate of about 10 nucleotides per 15 seconds and a broad distribution of DNA fragments [27]. These data indicate that bimolecular association of enzyme and DNA is a key step and suggest that simultaneous dilution of enzyme and DNA, whilst maintaining a stoichiometric excess of T4P, may offer a path to attenuated digestion rates by wild-type T4P. As a drawback, dilution of the DNA will disadvantage subsequent annealing of complementary single-stranded overhangs. In contrast, the digestion rates by T4P are not very sensitive to temperature. In one experiment, lowering the temperature from 25 °C to 10 °C reduced the digestion rate only about three-fold [6].

Available structural information suggests that the rate-limiting barrier presented by the initial digestion steps of blunt-ended DNA may more easily be crossed by the E2 mutant than wild-type T4P. The exonuclease and polymerase sites of T4P are in separate locations, where the polymerase site binds the primer/template DNA duplex and DNA binding to the proofreading exonuclease site requires strand separation of the last three nucleotides of the primer from the template to reach the exonuclease site [28]. The co-crystal structure of the construct N388 of the N-terminal domain of T4 DNA polymerase with p(dT)_3_ shows that the residue mutated in the E2 enzyme, Tyr320, is located near the exonuclease site and that the p(dT)_3_ oligonucleotide at the exonuclease site assumes an orientation that does not allow pairing with a complementary strand [29]. In the crystal structure, the side chain of Tyr320 is buried (although homologies with the Klenow fragment suggest that the side chain of Tyr320 can also assume a very different conformation and directly interact with DNA, indicating considerable flexibility of the peptide chain in which this residue is located [30]). As the empty space left behind by the mutation of Tyr320 to alanine affects the structure and flexibility of the exonuclease site, it is interesting to speculate that this explains the loss of intrinsic activity while, at the same time, facilitating accommodation of blunt-ended DNA with only two, one or even no terminal base pairs dissociated into single strands.

Protocols other than SLIC exist for restriction-enzyme-free cloning. In particular, the Gibson assembly protocol [3] has become a widely used method for molecular cloning of inserts into plasmids. In this protocol, single-stranded DNA overhangs are produced by T5 exonuclease which is a 5’- rather than 3’-exonuclease. The reaction is performed at 50 °C in the presence of a DNA polymerase and a DNA ligase, relying on gradual deactivation of the T5 exonuclease owing to its heat lability. While the Gibson protocol is elegant for operating in a single isothermal step and delivering fully re-annealed vectors prior to transformation, the DNA segments are usually designed with long matching overlaps to achieve successful hybridization at 50 °C, requiring relatively long primers. In contrast, RQ-SLIC does not depend on an elevated temperature for hybridization and therefore can be conducted with shorter single-stranded DNA overhangs and, consequently, shorter primers. The requirement of fewer enzymes saves cost too. As a drawback, reliance on intracellular *E*. *coli* enzymes for re-ligation of the final vector necessarily also leads to exposure to intracellular exonuclease activity and, therefore, loss of DNA. This problem becomes more severe with increasing number of ligations required. In one example, we obtained only about 100 colonies in a three-segment assembly by our standard RQ-SLIC protocol, indicating that longer overlap sequences may be necessary to join multiple DNA fragments [2].

It has recently been demonstrated that the RecA-independent *E*. *coli in vivo* recombination system can ligate overlapping segments of PCR-amplified DNA into plasmids without the need for any enzyme other than the DNA polymerase used in the PCR reaction [30]. This protocol depends on high-competency cells and two rounds of PCR to dilute parental plasmid. Large numbers of colonies were obtained only with primer overlap lengths of at least 25 nucleotides and the variation in colony number was relatively large.

Regarding site-directed mutagenesis, commercial QuikChange kits are specified to achieve greater than 80% mutagenesis efficiency for a single mutation site [31,32], but the kits depend on proprietory components. Our protocol achieves at least the same efficiency, delivers more colonies containing correctly mutated DNA, and does not rely on any proprietory components. In our hands, not using an exonuclease in the mutagenesis protocol greatly increased the number of colonies containing parental plasmid, whereas parental plasmid is rarely found in colonies following the E2 protocol. This translates into cost savings in DNA sequencing. Due to the polymerase activity of the E2 enzyme, however, separation of the PCR products from nucleotide triphosphates prior to digestion with E2 is a necessary step. In our laboratory, the E2 protocol has successfully been used for mutagenesis in hundreds of reactions over the past four years and the correct product is routinely identified following sequencing of no more than two colonies.

As with SLIC, the generation of single-stranded overhangs allows the use of relatively short primer–primer overlaps in the mutagenesis protocol, whereas the original QuikChange protocol favours longer overlaps due to its reliance on *in vivo* recombination [19]. For example, we successfully performed a three-site mutation spanning 12 nt between the first and third mutation site with a forward primer of 42 nt and a reverse primer of 32 nt using the E2 protocol, whereas the QuikChange@II web site [33] recommends primers of 52 and 65 nt, respectively.

Unligated circular plasmids prepared with the E2 protocols successfully transform cells of a competency as low as 10^6^ colony forming units per microgram DNA, which can readily be achieved by standard in-house techniques. With regard to the increased transformation efficiencies required for the construction of plasmid libraries, it is an advantage that the E2 enzyme is active also in buffers of low ionic strength, as this allows using larger amounts of reaction mixtures in cell transformations performed by electroporation.

## Conclusion

SLIC and QuikChange methods are convenient for their independence from restriction sites, by relying on endogenous DNA polymerase and ligase in *E*. *coli* to fill in and ligate the modified double-stranded plasmid after transformation. The present work shows that the success of both methods can be enhanced, without relying on proprietory components of costly kits, by using the 3’-exonuclease activity of the Y320A mutant of T4 DNA polymerase to generate single-stranded DNA overhangs. The attenuated activity of this mutant delivers uniform digestion rates of DNA under more convenient conditions of temperature and timing than the wild-type enzyme. The resulting improved control of enzymatic activity enhances the reliability of existing cloning and mutagenesis protocols and translates into corresponding savings in time and expense.

## Supporting information

S1 File Oligonucleotide primers, production of T4P mutants, digestion experiment of 100 bp DNA, multiple-site PCR, oligonucleotide primers used for site-directed mutagenesis of aminoacyl-tRNA synthetase. (PDF)
