# Supplementary material for: Mutant T4 DNA polymerase for easy cloning and mutagenesis
Source: PLoS One. 2019 Jan 23;14(1):e0211065. doi: 10.1371/journal.pone.0211065 (PMC6343910; doi:10.1371/journal.pone.0211065)
Supplement: S1 File — (PDF) [file pone.0211065.s001.pdf]

S1 File

Table A. Oligonucleotide primers used in this work.[a]

Name	Sequence
N388-r	ACAGAGAGAATTCAAAAGCTTTGTTTCACGTGAGAGCCCTG
E114A-f	CTGTGACATCGCAGTGACGGGGGATAAATTC
E114A-r	CCCGTCACTGCGATGTCACAGTTCGCTAC
Y320A-f	CTATATTTCCGCAAACATTATCGACGTAGAAAGCGTG
Y320A-r	GTCGATAATGTTTGCGGAAATATAGCGCTGATGGTTC
PET3[b]	CGACTCACTATAGGGAGACCACAAC
PET4[b]	CCTTTCGGGCTTTGTTAGCAG
seq-r[b]	GAATAATGGACGGGTAGAGAG
seq-f[b]	CTGGAAAGTGTAGCACAGCAC
v-f[c]	GAATTCGAGCTCCCGGGTAC
v-r[c]	CATATGTATATCTCCTTCTTAAAGTTAAAC
98bp-f	TCGATCCCGCGAAATTAATACG
8nt-f	TACATATGAAAGAATTTTATATCAGC
8nt-r	TCGAATTCAACCAAAC
15nt-f	GGAGATATACATATGAAAGAATTTTATATC
15nt-r	CGGGAGCTCGAATTC
20nt-f	AAGAAGGAGATATACATATGAAAGAATTTTATATC
20nt-r[d]	GTACCCGGGAGCTCG
25nt-f	ACTTTAAGAAGGAGATATACATATGAAAG
25nt-r[d]	CCATGGTACCCGGGAG

[a] All primers were ordered from Integrated DNA Technologies (Skokie, IL, USA). The characters f and r at the end of a primer name identify forward and reverse primers, respectively. Red characters indicate mutated codons. In primers used for insert amplification, segments matching the vector sequence are highlighted by underlining.

[b] Primers used for sequencing.

[c] Primers used for vector linearization.

[d] These primers were shorter than 20 and 25 nt, respectively, to match the melting temperatures of the corresponding forward primers.

Text A. Production of T4P mutants

The gene of full-length T4 DNA polymerase (T4P) was synthesised by Genscript (New Jersey, USA). Q5 high-fidelity DNA polymerase from New England Biolabs (Ipswich, MA, USA) was used to amplify the truncated form of T4P, N388, from T4P in pETMCSI [1] following the manufacturer's recommendations and using the primers PET3 and N388-r in Table A. PCR cycling used: initial denaturation at 98 °C for 2 min, followed by 30 cycles of (98 °C for 20 sec, 60 °C for 20 sec, 72 °C for 1 min) and final extension at 72 °C for 5 min. The point mutants E114A and Y320A were made using restriction-ligation and overlap PCR with the primer pairs PET3/E114A-r, E114A-f/PET4, PET3/Y320A-r and Y320A-f/PET4, respectively.

The T4P mutants were expressed in *E. coli* BL21(DE3)pLysS. Cells were grown in LB medium at 37 °C until the cell culture reached OD_{600} 0.7-1.0. Overexpression was induced with 0.5 mM IPTG and the culture was kept at 18 °C overnight. The harvested cell pellet was resuspended in buffer A (20 mM Tris-HCl, pH 7.6, 1 mM DTT, 1 mM EDTA and 10% glycerol) containing, in addition, 0.5 mM phenylmethanesulfonyl fluoride and lysed in a French Press (SLM Instruments; GE Healthcare, IL, USA). The lysate was cleared by centrifugation at 42,000 *g* for 40 min at 4 °C. Cleared lysate was loaded onto a DEAE column of a FPLC system at 4 °C. The column was washed by 3 column volumes of buffer A, followed by a salt gradient over 5 column volumes of 0-60% buffer B (buffer A with 1 M NaCl) and finally with 2 column volumes of buffer B. The T4P mutants eluted at 10-25% buffer B. The protein fractions were dialysed two times against 2 L buffer C (20 mM potassium phosphate, pH 6.8, 1 mM DTT, 10% glycerol). The dialysed DEAE fractions were loaded onto a 30 mL phosphocellulose column (P11; Whatman, Maidstone, UK) and the protein was eluted in a 0-100% buffer D gradient (buffer C containing 1 M KCl) using the FPLC. Protein fractions were pooled and concentrated using a Millipore Amicon Ultra-15 centrifugal filter unit (molecular weight cut off 10 kDa; Merck, Lebanon, NJ, USA). The yield was 30 mg of purified protein per litre cell culture. Diluent A (New England Biolabs) was used to dilute concentrated enzyme.

Text B. Digestion experiment of 98-bp DNA

The 98-bp DNA fragment (Fig A) was produced from the pETMCSI plasmid by PCR amplification with the primer set 98bp-f and v-r (Table A) using Vent DNA polymerase (New England Biolabs, Ipswich, MA, USA). The digestion experiment with E2 enzyme was performed in 7 tubes, each containing 200 ng 98-bp DNA in 20 μL T4 buffer with 1 μL of 31 μM E2 stock solution. The reaction mixtures were kept in a 37 °C water bath for 0, 15, 30, 45, 60, 75 and 90 min, respectively, and the enzyme was inactivated by heating at 72 °C for 20 min. The digestion experiment with wild-type T4P was performed in 5 tubes, each containing 200 ng 98 bp DNA in 20 μL T4 buffer with either 5 units (0.6 μg; New England Biolabs) T4P added, or T4P added in 3-, 10-, 30-, or 100-fold dilution. The reaction mixtures were incubated at 16 ºC for 30 minutes. Afterwards the enzyme was inactivated by heating at 72 ºC for 20 min. In preparation of analysis by PAGE, 400 μL H_2O and 400 μL of phenol/chloroform/isopropanol mixture (25 volume parts phenol, 24 parts chloroform, 1 part isopropanol; Sigma-Aldrich, St. Louis, MO, USA) were added to each tube, the mixture was agitated for 2 min and spun down at 11,000 *g* for 5 min. The supernatant was transferred to a new tube and 400 μL of chloroform/isopropanol mixture (24 parts chloroform, 1 part isopropanol) were added. The mixture was agitated and spun down as before. The cleared supernatant was transferred to a new tube and lyophilized. 20 μL formamide and 5 μL 6 x loading dye were added to each tube and the samples loaded onto a 12% denaturing acrylamide gel. The marker lane contained a mixture of available oligonucleotide primers of various size, each at a concentration of 100 ng/10 μL. The 12% continuous denaturing acrylamide gel was cast using degassed acrylamide solution in TBE buffer (89 mM Tris-borate, pH 8.3, 2 mM EDTA) containing 7 M urea and run at 10 mA constant current with running hot water heating the jacket chamber (around 50 °C). The gel was stained in 10 mL TBE buffer containing 2 μL SyBR Gold (Thermo Fisher Scientific, Waltham, MA, USA) for 2 h with agitation on an orbital shaker at room temperature.

```
TCGATCCCGC GAAATTAATA CGACTCACTA TAGGGAGACC ACAACGGTTT CCCTCTAGAA
ATAATTTTGT TTAACTTTAA GAAGGAGATA TACATATG
```

Figure A. Nucleotide sequence of the 98 bp DNA fragment used for the experiments of Fig 4.

Text C. Multiple-site mutagenesis PCR

18 different mutants of an aminoacyl-tRNA synthetase (evolved to incorporate *p*-cyanophenylalanine [2]) were produced by students of a third-year undergraduate course. Two different templates in pETMCSIII [1] were provided, pETMCSIII-SAYKQGK and pETMCSIII-SAYKQSK, which contained the mutations L32S/V65A/V103Y/W108K/M109Q/L162K and L32S/V65A/V103Y/W108K/M109Q/G158S/L162K, respectively. In each, the three residues Y103, K108, Q109 (YKQ) were to be mutated singly, doubly or triply, generating the 9 mutant combinations TKQ, TRK, YRK, TAQ, TRN, YRN, TKN, YKN and YQQ. For the two templates, this was achieved with the 18 forward and 2 reverse primers listed in Table B. The reverse primer T103-r was paired with each of the TXX primers and the reverse primer Y103-r was paired with the YXX primers (where the first X stands for either K, R or A, and the second X stands for K, Q or N). The reverse primers were designed to afford a melting temperature of 49.5 °C with the template, counting all nucleotides 3’ of the mutation mismatch and using the website Multiple Primer Analyzer [3] to calculate the melting temperature. By the same criterion, the melting temperature of the forward primers is 51.2 °C. The actual melting temperature was higher, as nucleotides 5’ of the mismatch also contribute, so that the actual melting temperature is estimated to be at least 10 °C higher. Whereas the forward mutation primers covered all three mutation sites, the reverse primer covers only one mutation site, which lies in the overlap region of the forward and reverse primers. The calculated melting temperature of the primer overlap is 45.3 °C. The PCR used 50 ng of template DNA and Q5 DNA polymerase, following the standard protocol recommended by the manufacturer (New England Biolabs). PCR protocol used: initial denaturation at 98 °C for 2 min, followed by 30 cycles of (98 °C for 20 sec, 60 °C for 20 sec, 72 °C for 3 min) and final extension at 72 °C for 5 min. The PCR product was purified using a PCR purification kit (Wizard SV gel and PCR clean-up system; Promega, Wisconsin, USA).

Table B. Oligonucleotide primers used for site-directed mutagenesis of aminoacyl-tRNA synthetase.

Name	Sequence
103T-r	TTCACTTCCATAGGTATATTTTGCCTTTAACC
103Y-r	TTCACTTCCATAATAATATTTTGCCTTTAACC
TKQG	ACCTATGGAAGTGAAAAACAGCTTGATAAGGATTATACACTG
TRKG	ACCTATGGAAGTGAACGTCAGCTTGATAAGGATTATACACTG
YRQG	TATTATGGAAGTGAACGTCAGCTTGATAAGGATTATACACTG
TAQG	ACCTATGGAAGTGAAGCGCAGCTTGATAAGGATTATACACTG
TRNG	ACCTATGGAAGTGAACGTAACCTTGATAAGGATTATACACTG
YRNG	TATTATGGAAGTGAACGTAACCTTGATAAGGATTATACACTG
TKNG	ACCTATGGAAGTGAAAAAAACCTTGATAAGGATTATACACTG
YKNG	TATTATGGAAGTGAAAAAAACCTTGATAAGGATTATACACTG
YQQG	TATTATGGAAGTGAACAGCAGCTTGATAAGGATTATACACTG
TKQS	ACCTATGGAAGTGAAAAACAGCTTGATAAGGATTATACACTG
TRKS	ACCTATGGAAGTGAACGTCAGCTTGATAAGGATTATACACTG
YRQS	TATTATGGAAGTGAACGTCAGCTTGATAAGGATTATACACTG
TAQS	ACCTATGGAAGTGAAGCGCAGCTTGATAAGGATTATACACTG
TRNS	ACCTATGGAAGTGAACGTAACCTTGATAAGGATTATACACTG
YRNS	TATTATGGAAGTGAACGTAACCTTGATAAGGATTATACACTG
TKNS	ACCTATGGAAGTGAAAAAAACCTTGATAAGGATTATACACTG
YKNS	TATTATGGAAGTGAAAAAAACCTTGATAAGGATTATACACTG
YQQS	TATTATGGAAGTGAACAGCAGCTTGATAAGGATTATACACTG

[a] All primers were ordered from Integrated DNA Technologies. The character r at the end of a primer name identifies reverse primers. The characters G and S at the end of a primer name identifies the sets of forward primers for pETMCSIII-SAYKQGK and pETMCSIII-SAYKQSK, respectively. Mismatches with respect to the template are underlined. The mutation site located in the overlap region of the forward and reverse primers is highlighted in red.

References

1. Neylon C, Brown SE, Kralicek AV, Miles CS, Love CA, Dixon NE. Interaction of the *Escherichia coli* replication terminator protein (Tus) with DNA: a model derived from DNA-binding studies of mutant proteins by surface plasmon resonance. Biochemistry. 2000; 39: 11989–11999.
2. Young DD, Young TS, Jahnz M, Ahmad I, Spraggon G, Schultz PG. An evolved aminoacyl-tRNA synthetase with atypical polysubstrate specificity. Biochemistry. 2011; 50: 1894–1900.
3. https://www.thermofisher.com/au/en/home/brands/thermo-scientific/molecular-biology/molecular-biology-learning-center/molecular-biology-resource-library/thermo-scientific-web-tools/multiple-primer-analyzer.html, accessed 28 August 2018.
